# Maxillomandibular advancement for obstructive sleep apnea: a retrospective prognostic factor study for surgical response

**DOI:** 10.1007/s11325-022-02731-x

**Published:** 2022-10-22

**Authors:** Ning Zhou, Jean-Pierre T. F. Ho, Wouter P. Visscher, Naichuan Su, Frank Lobbezoo, Jan de Lange

**Affiliations:** 1 Amsterdam UMC Location University of Amsterdam, Department of Oral and Maxillofacial Surgery, University of Amsterdam, Meibergdreef 9, Amsterdam, 1105 AZ The Netherlands; 2 Academic Centre for Dentistry Amsterdam (ACTA), University of Amsterdam and Vrije Universiteit Amsterdam, Amsterdam, The Netherlands; 3 Department of Orofacial Pain and Dysfunction, Academic Centre for Dentistry Amsterdam (ACTA), University of Amsterdam and Vrije Universiteit Amsterdam, Amsterdam, The Netherlands; 4 Department of Oral and Maxillofacial Surgery, Northwest Clinics, Alkmaar, The Netherlands; 5 Department of Oral Public Health, Academic Centre for Dentistry Amsterdam (ACTA), University of Amsterdam and Vrije Universiteit Amsterdam, Amsterdam, The Netherlands

**Keywords:** Obstructive sleep apnea, Maxillomandibular advancement, Surgical response, Predictor

## Abstract

**Purpose:**

To identify potential predictors of surgical response to maxillomandibular advancement (MMA) in patients with obstructive sleep apnea (OSA) from the most common clinically available data (patient-related, polysomnographic, cephalometric, and surgical variables).

**Methods:**

This was a retrospective study comprised of consecutive patients who underwent MMA for moderate to severe OSA. Relevant clinical, polysomnographic, cephalometric, and surgical variables were collected as independent variables (predictors). The association of the independent variables with a favorable surgical response to MMA was assessed in univariate and multivariate analyses.

**Results:**

In 100 patients (82% male; mean age 50.5 years), the mean apnea hypopnea index [AHI] was 53.1 events/h. The rate of favorable surgical response was 67%. Based on multivariate analysis, patients with cardiovascular disease (CVD) had 0.140 times lower odds of a favorable response to MMA (OR: 0.140 [0.038, 0.513], *P* = 0.003). For each 1-unit increase in central apnea index (CAI) and superior posterior airway space (SPAS), there were 0.828 and 0.724 times lower odds to respond favorably to MMA (OR: 0.828 [0.687, 0.997], *P* = 0.047; and 0.724 [0.576, 0.910], *P* = 0.006), respectively.

**Conclusion:**

The findings of this study suggest that the surgical outcome of MMA may be less favorable when patients with OSA have certain phenotypic characteristics: the presence of CVD, higher CAI and larger SPAS. If confirmed in future studies, these variables may guide patient selection for MMA.

**Supplementary information:**

The online version contains supplementary material available at 10.1007/s11325-022-02731-x.

## Introduction

Maxillomandibular advancement (MMA) is a skeletal surgery for treatment of obstructive sleep apnea (OSA) MMA enlarges the upper airway space and reduces the upper airway collapsibility by displacing the maxilla and mandible anteriorly [1, 2]. Despite the fact that MMA has been demonstrated to be a highly effective therapy for moderate to severe OSA, with a surgical success rate of approximately 85% [3, 4], there are still patients who do not respond as favorably as others to MMA. In order to improve preoperative counselling of patients regarding the chance of surgical response, and also to avoid ineffective therapy and unnecessary burden on non-responders to MMA, it is essential and clinically meaningful to identify the potential responders and non-responders to MMA prior to the surgery.

Some factors have been reported to correlate with increased surgical response to MMA, mainly in terms of patient-related characteristics, polysomnographic variables, and surgical characteristics. For example, a meta-analysis suggested that younger age, lower baseline weight, lower baseline apnea hypopnea index [AHI], and greater degree of maxillary advancement were associated with increased surgical response [4]. In addition, a few studies also identified radiographic or drug-induced sleep endoscopy (DISE) predictors of surgical response to MMA [5–7], such as cephalometric minimum retrolingual space [6] and complete anteroposterior epiglottic collapse during DISE [7]. However, the evidence on predictors of MMA surgical outcome remains incomplete. Consequently, the clinicians’ ability to predict MMA outcome and pre-select suitable candidates for MMA is limited and is based mainly on the clinician’s expertise.

For patients undergoing MMA for OSA, a preoperative assessment in daily clinical practice mainly involves medical and sleep history, physical and radiographic examination, a polysomnography (PSG), and sometimes a DISE. Therefore, the aim of this study was to identify the potential predictors of surgical response to MMA in patients with OSA, from the most common clinically available data (patient-related, polysomnographic, cephalometric, and surgical variables).

## Methods and materials

### Patient selection

This study recruited consecutive patients who underwent MMA for OSA at the Department of Oral and Maxillofacial Surgery, Amsterdam UMC (location AMC), from September 2011 to July 2021. The further inclusion criteria were the following: (1) age 18 years or older; (2) the presence of moderate to severe OSA diagnosed by an overnight PSG; (3) continuous positive airway pressure (CPAP) failure, intolerance, or refusal; and (4) patients with a follow-up PSG recording at least three months after MMA. The exclusion criteria were as follows: (1) patients who declined their data to be used for research purposes; (2) previous history of a LeFort I osteotomy and/or a bilateral sagittal split osteotomy (BSSO); and (3) craniofacial and/or syndromic patients.

### Variables

All data were retrospectively collected from patients’ electronic files. Recorded baseline characteristics included patient-related variables, respiratory variables as measured by PSG, and cephalometric variables. Postoperative PSG variables and cephalometric measurements were also recorded. The surgical characteristics were determined by preoperative and postoperative cephalograms. The potential predictors of MMA surgical response included the recorded baseline characteristics and surgical characteristics.

### Patient-related variables

The collected patient-related variables included age, gender, body mass index (BMI), preoperative physical status represented by the ASA (American Society of Anesthesiology) classification system score [8], specific comorbidities (i.e., hypertension, cardiovascular diseases [CVD] [9], diabetes mellitus, and chronic obstructive pulmonary disease), previous history of upper airway surgery for OSA, and the number of lost teeth. The tooth loss was categorized as the following: 0–4 lost teeth, 5–8 lost teeth, 9–31 lost teeth, and 32 lost teeth, i.e., being edentulous [10].

### Polysomnography

An overnight PSG was performed preoperatively and at least 3 months postoperatively. All respiratory events were scored according to the American Academy of Sleep Medicine (AASM) criteria [11]. The collected baseline PSG variables included AHI, central apnea index (CAI), mixed apnea index (MAI), positional OSA or non-positional OSA (positional OSA was defined as an AHI at least twice as high in supine position as in non-supine position [12]), 3% oxygen desaturation index (3% ODI), and lowest oxygen saturation (LSAT).

Postoperative AHI, 3% ODI, and LSAT were collected to assess the surgical outcome. According to Sher’s criteria, surgical response was defined as “at least 50% AHI reduction following MMA and a postoperative AHI < 20” [13].

### Cephalometry

All patients underwent a standardized lateral cephalogram preoperatively and at least one week postoperatively. All radiographs were taken with the subjects in natural head position with centric occlusion and lips at rest. Cephalometric analysis was performed by one observer using Viewbox software (Viewbox 4, dHAL Software, Kifissia, Greece). Twenty-two cephalometric variables for skeletal and soft tissue, including the cranial base, face height, maxilla and mandible, soft palate, tongue, hyoid, and upper airway, were measured (Table 1; Supplementary e-Fig. 1 and e-Fig. 2).

**Table 1:** Overview of cephalometric variables and definitions

	Variable	Definition
Cranial base	S–N	Distance between S and N
	N-S-Ba	Angle from N to S to Ba
Face height	ATFH	Distance between N and Me
	ALFH	Distance between ANS and Me
	PTFH	Distance between S and Go
	MP-SN	Inclination of the mandibular plane in relation to the SN plane
Maxilla and mandible	SNA	Angle from S to N to A
	SNB	Angle from S to N to B
	ANB	Angle from A to N to B
	Maxillary length	Distance between ANS and PNS
	Mandibular corpus length	Distance between Go and Me
Soft palate	SPL	Distance between PNS and UT
	SPT	Maximal diameter of soft palate perpendicular to PNS-UT line
Tongue	TGL	Tongue length as the distance between TT and Eb
	TGH	Maximum tongue height perpendicular to TT-Eb line
Hyoid bone	H–S	Distance between H and S
	H-MP	Distance between H and MP
	H-C3	Distance between H and C3
Upper airway	UAL	Upper airway length as distance between PNS to Eb
	SPAS	Width of airway along parallel line to Go-B line at the level of the midpoint of UT and PNS
	MAS	Width of airway along parallel line to Go-B line through UT
	IAS	Width of airway along Go-B line
Surgical movement	A-TVP	Distance between A to TVP
	B-TVP	Distance between B to TVP
	Pog-TVP	Distance between Pog to TVP

A, A-point (subspinale); ALFH, anterior lower face height; ANS, anterior nasal spine; ATFH, anterior total face height; B, B-point (supramentale); Ba, basion; C3, the most anterior-inferior point of the third cervical vertebra; Eb, epiglottis base; Go, gonion; H, hyoid point; IAS, inferior airway space; MAS, middle airway space; Me, menton; MP, mandibular plane; N, nasion; PNS, posterior nasal spine; Pog, pogonion; PTFH, posterior total face height; S, sella; SN, sella-nasion line; SPAS, superior posterior airway space; SPL, soft palate length; SPT, soft palate thickness; TGH, tongue height; TGL, tongue length; UAL, upper airway length; UT, uvula tip; TT, tongue tip; TVP, true vertical plane

To quantify the reliability of the measurements, the same observer repeated the tracings in 20 randomly selected radiographs one month later.

### Maxillomandibular advancement

The MMA procedures were completed by two dedicated OSA surgeons and consisted of a LeFort I osteotomy of the maxilla and a BSSO of the mandible. The maxillomandibular complex was advanced and counterclockwise rotation was performed for selected cases. The surgical variables used in this study included degrees of A-point, B-point and pogonion (Pog) advancement, and presence or absence of anticlockwise rotation. The degrees of A-point, B-point, and Pog advancement were determined by comparing preoperative and postoperative distance between A-point to the true vertical plane (TVP), B-point to TVP, and Pog to TVP, respectively. After MMA, cases with a mandibular plane angle change of ≤ -2 degrees were classified as counterclockwise rotation cases [14].

### Statistical analysis

All collected data were analyzed with SPSS (IBM SPSS Statistical version 26, IBM Corp., Armonk, NY, USA). Normality was tested using the Shapiro–Wilk test. Continuous variables were reported as mean and standard deviation when normally distributed or as median and interquartile range when not normally distributed. Categorical variables were reported as frequency and percentage. To compare the preoperative and postoperative continuous variables, the paired-samples t-test or Wilcoxon signed-rank test was applied in cases of normally or non-normally distributed data, respectively. To compare the continuous variables between responders and non-responders, the independent-samples t-test or Mann–Whitney *U* test was used in cases of normally or non-normally distributed data, respectively. Chi-square test was used to compare the categorical variables between responders and non-responders. The intra-observer reliability of the cephalometric measurements was evaluated using intraclass correlation coefficient (ICC).

Logistic regression was used to identify the variable(s) that was (were) predictive of a favorable response to MMA. First, univariate logistic regression analyses were used to assess the association between each independent variable (predictor) and the surgical response, separately. Multivariate logistic regression with backward selection (*P* < 0.05 for removal) was then used to identify the variables that were independently associated with the surgical response. The independent variables included in the multivariate model were those with a *P* value of < 0.10 in univariate logistic regression. For variables including age, gender, BMI, baseline AHI, and degrees of maxillary and mandibular advancement, they were forced into the multivariate model regardless of their *P* values in univariate logistic regression because of their potential importance for MMA surgical outcome [4]. Collinearity diagnostics test was performed using the variance inflation factors (VIF) cut-off value of 5; a variable(s) with VIF greater than 5 was excluded from the multivariate model. Complete case analysis was used to handle the missing values for logistic analysis. A *P* value < 0.05 was considered statistically significant.

## Results

### Patient characteristics

A total of 111 patients underwent MMA for obstructive sleep apnea (OSA). Of these, 100 patients (82% male) were included in this study. The reasons for exclusion from the study were as follows: no follow-up PSG available (*n* = 4), rejected their data to be used for research (*n* = 3), mild OSA (*n* = 3), and craniofacial and/or syndromic patient (*n* = 1). Participants were middle aged (50.5 ± 9.9 years) and overweight (BMI = 29.8 ± 4.2 kg/m^2^), with a mean baseline AHI of 53.1 ± 21.2 events/h.

### Surgical outcome

The mean degrees of A-point, B-point, and Pog advancement were 7.2 ± 2.3 mm, 9.8 ± 4.2 mm, and 9.8 ± 5.1 mm, respectively. The postoperative PSGs were performed 4.0 (3.0–6.0) months after MMA. At the time of postoperative PSG, the mean BMI of the patients was 29.1 ± 4.5 kg/m^2^. The major outcomes of the MMA surgery in the total population are shown in Table 2. The median AHI was significantly reduced from 51.7 (36.8–68.5) events/h to 12.9 (5.9–23.1) events/h (*P* < 0.001). A favorable surgical response was achieved in 67 of 100 patients (67%), and 19 patients (19%) had an AHI of < 5 events/h postoperatively. The preoperative and postoperative PSG values and upper airway measurements in responders and non-responders are presented in Supplementary e-Table 1.

**Table 2:** Treatment outcome of maxillomandibular advancement in the total population

Variable	Preoperative (*n* = 100)	Postoperative (*n* = 100)	*P*
AHI (events/h)	51.7 (36.8–68.5)	12.9 (5.9–23.1)	< 0.001*
ODI 3% (events/h)	51.0 (34.3–66.6)	21.2 (10.5–30.2)	< 0.001*
LSAT (%)	79.5 (73.0–84.0)	86.0 (82.0–89.0)	< 0.001*

Data presented as median (Q1–Q3). AHI, apnea hypopnea index; LSAT, lowest oxygen saturation; n, number of patients; ODI 3%, 3% oxygen desaturation index

^*^Statistically significant difference preoperative versus postoperative values (*P* value < 0.05)

### Baseline and surgical characteristics and surgical response

Compared to responders, the occurrences of hypertension and CVD were significantly higher in non-responders (*P* = 0.003 and 0.001, respectively). Preoperative CAI was significantly higher in non-responders (*P* = 0.011) (Table 3). ICC of the cephalometric analysis ranged from 0.859–0.998, which indicated an excellent intra-observer reliability [15]. Of the cephalometric variables, non-responders had a significantly larger superior-posterior airway space (SPAS; *P* = 0.002) than responders (Table 4). There were no significant differences between responders and non-responders in the other baseline characteristics. In terms of surgical characteristics, no significant difference was found between responders and non-responders (Table 4).

**Table 3:** Patient-related variables and polysomnographic variables in responders and non-responders

Variable	Responder (*n* = 67)	Non-responder (*n* = 33)	*P*
Patient-related variables			
Age (years)	49.0 (41.0–59.0)	54.0 (45.5–58.0)	0.162
Male (*n*, %)	54 (81)	28 (85)	0.603
BMI (kg/m^2^)	29.7 (27.4–32.4)	29.8 (28.2–32.0)	0.652
ASA score			
I	17 (25%)	6 (18%)	0.487
II	38 (57%)	18 (55%)	
III	12 (18%)	9 (27%)	
Hypertension (*n*, %)			
Absence	49 (73)	14 (42)	0.003*
Presence	18 (27)	19 (58)	
CVD (*n*, %)			
Absence	51 (76)	14 (42)	0.001*
Presence	16 (24)	19 (58)	
DM (*n*, %)			
Absence	58 (87)	29 (88)	1.000
Presence	9 (13)	4 (12)	
COPD (*n*, %)			
Absence	64 (96)	31 (94)	1.000
Presence	3 (5)	2 (6)	
Previous upper airway surgery (*n*, %)			
Absence	40 (60)	20 (61)	0.931
Presence	27 (40)	13 (39)	
Lost teeth (*n*, %)			
0–4 lost teeth	15 (22)	4 (12)	0.527
5–8 lost teeth	28 (42)	13 (39)	
9–31 lost teeth	16 (24)	10 (30)	
32 lost teeth	8 (12)	6 (18)	
Polysomnographic variables			
AHI (events/h)	54.2 ± 20.9	50.9 ± 21.9	0.474
CAI (events/h)	0.4 (0.2–1.4)^a^	1.5 (0.4–6.3)^b^	0.011*
MAI (events/h)	1.9 (0.2–9.1)^a^	5.6 (0.8–14.6)^b^	0.129
Positional/non-positional OSA (n, %)			
Positional OSA	22 (43)	11 (38)	0.649
Non-positional OSA	29 (57)	18 (62)	
ODI 3% (events/h)	52.4 ± 22.3	51.5 ± 21.0	0.866
LSAT (%)	79 (71.0–84.0)	80 (76.0–85.0)	0.236

Continuous data presented as mean ± standard deviation or median (Q1–Q3), categorical data presented as number with percentage. AHI, apnea hypopnea index; ASA, American Society of Anesthesiology; BMI, body mass index; CAI, central apnea index; COPD, chronic obstructive pulmonary disease; CVD, cardiovascular disease; DM, diabetes mellitus; LSAT, lowest oxygen saturation; MAI, mixed apnea index; n, number of patients; ODI 3%, 3% oxygen desaturation index; OSA, obstructive sleep apnea

^*^Statistically significant difference responders versus non-responders (*P* value < 0.05)

^a^Number of patients = 55

^b^Number of patients = 29

**Table 4:** Cephalometric variables and surgical variables in responders and non-responders

Variable	Responder	Non-responder	*P*
Cephalometric variables (responder: *n* = 64; non-responder: *n* = 31)			
Cranial base			
S–N (mm)	70.0 ± 3.6	70.6 ± 4.0	0.408
N-S-Ba (degree)	130.0 (126.5–132.2)	131.2 (128.2–134.5)	0.076
Face height			
ATFH (mm)	122.0 ± 7.9	124.5 ± 9.7	0.197
ALFH (mm)	71.6 ± 7.0	73.7 ± 8.3	0.213
PTFH (mm)	80.0 ± 7.9	82.2 ± 8.1	0.222
MP-SN (degree)	36.7 ± 8.4	36.7 ± 10.9	0.979
Maxilla and mandible			
SNA (degree)	80.3 ± 3.6	80.0 ± 4.0	0.760
SNB (degree)	75.1 ± 4.1	76.5 ± 4.7	0.149
ANB (degree)	5.2 ± 2.6	3.7 ± 4.2	0.093
ANS-PNS (mm)	53.0 ± 3.4	52.8 ± 4.2	0.745
Go-Me (mm)	65.0 ± 6.4	66.7 ± 5.9	0.226
Soft palate			
SPL (mm)	39.6 ± 7.0	40.4 ± 5.9	0.561
SPT (mm)	9.9 (8.6–11.4)	11.0 (9.4–11.9)	0.096
Tongue			
TGL (mm)	84.0 (79.8–87.3)	83.7 (79.7–88.1)	0.795
TGH (mm)	36.5 ± 3.9	35.2 ± 4.6	0.156
Pharyngeal dimensions and hyoid bone position			
UAL (mm)	76.8 ± 6.4	78.8 ± 7.7	0.249
SPAS (mm)	7.3 (5.5–9.2)	8.8 (7.6–11.0)	0.002*
MAS (mm)	9.9 ± 2.6	10.8 ± 3.4	0.172
IAS (mm)	8.9 ± 3.1	9.2 ± 3.0	0.625
H–S (mm)	118.0 ± 9.5	120.7 ± 9.6	0.197
MP-H (mm)	25.4 ± 5.5	25.9 ± 5.9	0.682
H-C3 (mm)	39.4 ± 4.8	41.4 ± 6.7	0.105
Surgical variables (responder: *n* = 63; non-responder: *n* = 29)			
Advancement degree of A-point (mm)	7.0 ± 2.5	7.4 ± 1.9	0.485
Advancement degree of B-point (mm)	10.0 ± 4.3	9.6 ± 4.0	0.678
Advancement degree of Pog (mm)	9.8 ± 5.2	9.9 ± 5.2	0.909
Counterclockwise rotation (n, %)			
Absence	29 (46.0)	10 (34.5)	0.298
Presence	34 (54.0)	19 (65.6)	

Continuous data presented as mean ± standard deviation or median (Q1–Q3), categorical data presented as number with percentage. A, A-point; ALFH, anterior lower face height; ANS, anterior nasal spine; ATFH, anterior total face height; B, B-point; Ba, basion; C3, the most anterior-inferior point of the third cervical vertebra; Go, gonion; H, hyoid bone; IAS, inferior airway space; MAS, middle airway space; Me, menton; mm, millimeter; MP, mandibular plane; N, nasion; n, number of patients; PNS, posterior nasal spine; PTFH, posterior total face height; S, sella; SPL, soft palate length; SPAS, superior posterior airway space; SPT, soft palate thickness; TGL, tongue length; TGH, tongue height; UAL, upper airway length

^*^Statistically significant difference responders versus non-responders (*P* value < 0.05)

### Prediction of surgical response

The univariate analyses revealed six independent variables with a *P* value < 0.1 (Supplementary e-Table 2). After collinearity diagnostics test, all the six variables were included in the multivariate model, including age, hypertension, CVD, CAI, ANB, and SPAS (Table 5).

**Table 5:** Logistic regression model for predicting surgical response to maxillomandibular advancement

Univariate analysis					Multivariate analysis (adjusted for the covariables: gender, BMI, AHI, advancement of A-point, and advancement of B-point)			
Independent variable	Coefficient B	SE	OR (95%CI)	*P*	Coefficient B	SE	OR (95%CI)	*P*
Age	− 0.041	0.023	0.959 (0.917, 1.003)	0.070				
Hypertension								
Absence	Ref							
Presence	− 1.307	0.447	0.271 (0.113–0.650)	0.003				
CVD								
Absence	Ref				Ref			0.003
Presence	− 1.465	0.454	0.231 (0.095–0.563)	0.001	− 1.964	0.662	0.140 (0.038–0.513)	
CAI	− 0.191	0.080	0.826 (0.707–0.966)	0.017	− 0.189	0.095	0.828 (0.687, 0.997)	0.047
ANB	0.144	0.074	1.155 (1.000–1.334)	0.051				
SPAS	− 0.242	0.083	0.785 (0.666–0.924)	0.004	− 0.323	0.117	0.724 (0.576–0.910)	0.006

ANB, angle from A-point to nasion to B-point; CAI, central apnea index; CI, confidence interval; CVD, cardiovascular disease; OR, odds ratio; Ref., reference category; SE, standard error; SPAS, superior posterior airway space

After adjusting for the covariables (gender, BMI, AHI, and degrees of maxillary and mandibular advancement), the multivariate model revealed that the independent factors associated with surgical response were CVD, CAI, and SPAS. Patients with the presence of CVD had 0.140 times lower odds to respond favorably to MMA (OR: 0.140 [0.038, 0.513]; *P* = 0.003) compared with those without. For each 1-unit increase in CAI, there was 0.828 times lower odds to respond favorably to MMA (OR: 0.828 [0.687, 0.997]; *P* = 0.047). For each 1-unit increase in SPAS, there was 0.724 times lower odds to respond favorably to MMA (OR: 0.724 [0.576, 0.910]; *P* = 0.006).

## Discussion

The present study aimed to investigate if the most common clinically available data, i.e., patient-related, polysomnographic, cephalometric, and surgical variables, have predictive value on MMA surgical outcome. Our main finding was that among baseline and surgical characteristics, cardiovascular disease (CVD), central apnea index (CAI), and superior posterior airway space (SPAS) were the independent predictors of response to MMA: the presence of CVD is indicative of non-response, and CAI and SPAS are inversely related to a favorable response.

Notably, in the present study, the overall success rate of MMA — 67% —was lower than that reported in previous studies [3], which ranged from 70 to 100%. One probable reason for this difference in the success rate between the present study and previous studies is that patients recruited in our institute for MMA have been refractory to multiple therapies (e.g., CPAP, mandibular advancement device, upper airway surgery), or were considered poor candidates for upper airway surgery for various reasons (e.g., central and mixed apneas > 25% of the total AHI [16], multilevel complete collapse during DISE [17]). Thus, for some of our patients, there could be a complex interplay between anatomical and non-anatomical traits in OSA pathogenesis, which might have led to the relatively low success rate in our study. In addition, although baseline DISE was not performed in all the patients, over half of the study population (65/100) received DISE, 52 of whom presented with epiglottic collapse. A recent study from Kastoer et al. suggested that MMA surgery may not be an effective therapy for epiglottic collapse [18].

Prior work has suggested that OSA is associated with CVD [19, 20]. In a recent study consisting of 1717 patients with moderate to severe OSA, the prevalence of CVD was 52% [20]. In the present study, CVD also affects 35% of our study population (26 patients with coronary heart disease, six patients with cerebrovascular disease, and three patients with both coronary heart disease and cerebrovascular disease; seven of these patients had heart failure), which further supports the notion that CVD is highly prevalent in patients with OSA. Notably, our study is the first to show that the presence of CVD in patients with OSA is independently associated with non-response to MMA. We inferred that OSA with coexisting CVD may represent a subtype involving a complex interaction between anatomical and non-anatomical causes of OSA that cannot be fully resolved by MMA. Currently, only very limited evidence can partially support our inference. It has been suggested that chronic hypoxemia and/or high left atrial pressure in heart failure could yield an elevated loop gain via increases in chemosensitivity [21]. Additionally, the increased fluid retention and nocturnal rostral fluid shift in heart failure could narrow the upper airway and increase the extraluminal tissue pressure [22]. In this study population, however, the post hoc chi-square test showed that there is no significant difference in the percentage of heart failure between responders and non-responders (6% (4/67) vs 9% (3/33), *P* = 0.874). Further work should be performed to investigate the underlying pathophysiological mechanism of OSA with coexisting CVD for personalized treatment. Additionally, it is important to take into account the duration of CVD for its severity and to use such severity as an element for subgrouping in order to investigate the contribution of CVD to the surgical outcome of MMA. However, among the 35 patients with CVD, the duration of CVD is only available in 7 patients (10.1 ± 3.9 years, range 6–16 years), which prevents us from further analysis of those patients. Future investigations are necessary to confirm our finding and to explore the association between duration of CVD and MMA surgical response.

In clinical practice, it is not uncommon that individuals with OSA exhibit some proportion of central and/or mixed events, leading to a dilemma in the selection of the most appropriate OSA treatment. Our study demonstrated that a higher preoperative CAI was independently associated with non-response to MMA. This finding is supported by a previous study by Makovey et al. [5], which found that the mean pre-MMA CAI in their failure group was significantly higher than that in their success group (5.7 events/h vs 0.6 events/h; *P* = 0.005). The heterogeneity of pure OSA (i.e., 100% of apneas are obstructive) and predominant OSA (i.e., coexisting obstructive and central apneas, and 50% < obstructive apneas < 100%) has been investigated previously [23]. It was suggested that the pure OSA group and predominant OSA group have equally elevated upper airway collapsibility (i.e., critical closing pressure [Pcrit]); however, the patients with predominant OSA differed from the patients with pure OSA in showing less breathing control stability [23]. The finding that patients with OSA and relatively higher baseline CAI are less likely respond favorably to MMA also indicates that in these patients breathing control instability may play a significant role in the development of obstructive events. Recently some studies have suggested that breathing control instability (high loop gain) promotes treatment failure on oral appliance or upper airway stimulation for patients with OSA [24–26]. Future research is required to determine whether or not treatment for central respiratory instability in patients with predominant OSA may help relieve the obstructive events.

So far, little evidence is available on the predictive value of cephalometric variables in terms of surgical response to MMA in patients with OSA. In this study, we have included parameters of craniofacial and upper airway morphology such as maxillary and mandibular position, face height, soft palate, and tongue, which have not been assessed together in previous studies on surgical response to MMA. This patient cohort presented only one cephalometric variable that is independently related to MMA surgical response, i.e., SPAS. We found that larger SPAS was independently associated with non-response to MMA. This finding is in line with that in a study by Teitelbaum et al. [6]. Their study showed that the minimal SPAS in their MMA success group was significantly narrower than that in their MMA failure group (4.6 ± 1.3 mm vs 7.2 ± 1.7 mm, *P* = 0.009). There are several possible explanations for our finding. First and foremost, in this study cephalograms were taken with the patients awake in upright position. Most of skeletal cephalometric parameters such as cranial base and mandibular length could completely reflected the condition during sleep as they are stable and independent of posture and sleep state, whereas the skeletal parameters that could be affected by mandibular movement (e.g., SNB, ANB) and soft tissue parameters (e.g., soft palate, pharyngeal space) might not. As a consequence, the value of SPAS, as well as some other cephalometric measures, in predicting surgical response to MMA might have been over- or underestimated. Secondly, it has been suggested that airway shape may be a predisposing factor for the development of OSA; patients with OSA are likely to have an elliptical airway with the long axis oriented anteroposteriorly (A-P), and this A-P orientation may adversely affect the airway muscle function which results in airway collapse during sleep [27]. We hypothesize that the patients with OSA and larger SPAS are more likely to present with A-P airway orientation. Several previous studies have shown that after MMA there were significant increases in both lateral and A-P airway diameters, and the ratio of A-P and lateral airway dimension tended to be higher [28, 29]. This indicates that MMA surgery may actually exacerbate the A-P airway orientation in some patients, leading to a less beneficial surgical outcome. Of note, MMA can not only alter the upper airway morphology, but also increase the pharyngeal wall tension [30]. The latter element, i.e., pharyngeal wall tension, was not evaluated and therefore not weighed in this study. Lastly, for patients with OSA and a larger pharyngeal airway space, there is a higher possibility that non-anatomical contributors play a more prominent role in the pathogenesis of OSA, which may not be treated with MMA. The predictive value of SPAS for MMA surgical outcome needs further investigation. Furthermore, the predictive value of 3D upper airway parameters (e.g., volume, cross-sectional area) should be also explored.

It is interesting to note that several other predictors recognized previously were found not to be predictive of surgical response in our study, mainly including lower baseline AHI, lower baseline BMI, and larger degree of maxillary advancement [4]. Currently, there is still a question as whether these factors could predict MMA surgical response. In a study from Goodday et al. [31], the efficacy of MMA was evaluated in 13 cases of OSA with an AHI higher than 100 events/h, and a favorable surgical response was achieved in 10 of those patients. The authors concluded that MMA was highly effective for patients with extremely severe OSA. Of note, although AHI is currently the most widely used measure of OSA severity, there is a growing recognition in its limitation to predict clinical consequences of OSA and response to OSA treatment [32]. Recently, some other alternative measures of OSA severity have been proposed, such as apnea–hypopnea event duration [33] and hypoxic burden [34]. However, our study did not analyze such PSG parameters because these relatively novel measures were not available in the PSG reports of our patients. Future research should explore the value of these alternative measures in predicting response to MMA. Besides, due to the fact that in the study by Goodday et al. [31], eight of nine patients with available BMI values were obese (BMI range 31.2–61.3 kg/m^2^) before surgery, and all but one remained obese (BMI range 29– 53.9 kg/m^2^) after surgery, they assumed that BMI did not appear to influence changes in AHI. With regard to the maxillary advancement, multiple studies have found no correlation between degree of maxillary advancement and a reduction in AHI [35, 36]. Increased airway volume following MMA has been considered to be necessary for improving OSA [28, 37], while Chang et al. reported that there was a plateau effect for the airway volume increase as a result of maxillary advancement [38]. In addition to the potential predictors mentioned above, some other factors of interest to clinicians were also investigated in terms of predicting MMA outcome. For example, tooth loss may be an independent risk factor for OSA [10], but few evidence is available on the association between the number of lost teeth and treatment outcome for OSA [39]. This study is the first to suggest that MMA outcome is not significantly related to number of lost teeth. Taken together, more research is required to recognize which parameters can reliably predict the surgical response, and thus should be included in the patient selection procedure of MMA for OSA.

The study results should be interpreted with caution due to certain limitations. First, it was a retrospective study, whereas a prospective study would allow for better control of the data. Second, our cohort consisted predominantly of middle-aged, overweight males with severe OSA, thus the results may be limited to this patient profile. Furthermore, as we have stated before, given those relatively novel PSG measures of OSA severity (e.g., hypoxic burden) were absent in PSG reports of our patients, such parameters were not included in the analysis. This may also limit the generalizability of our findings. Lastly, the cephalograms were obtained with the patient awake in a standard upright position. Some measurement results, especially the soft tissue measurements, may thus not represent the condition during sleep. This may explain why most of the measurements of upper airway structures cannot be implicated in the surgical outcome. However, from the aspects of cost and/or convenience, an upright cephalogram remains an important imaging technique to evaluate the craniofacial and upper airway anatomy.

## Conclusion

Within the limitations of the study, the findings suggest that the presence of cardiovascular disease, higher central apnea index, and larger superior posterior airway space are independently associated with non-response to MMA for OSA. Our results may further support the concept that OSA is a heterogeneous disorder with multifactorial pathophysiological causes, which highlights the importance of evolving different OSA phenotypes and thereby developing personalized treatment.

## Supplementary information

Below is the link to the electronic supplementary material.

Supplementary file1 (DOCX 861 KB)

## Data Availability

The datasets generated during and/or analyzed during the current study are available from the corresponding author on reasonable request.
