# Supplementary material for: Maxillomandibular advancement for obstructive sleep apnea: a retrospective prognostic factor study for surgical response
Source: Sleep Breath. 2022 Oct 22;27(4):1567–76. doi: 10.1007/s11325-022-02731-x (PMC10427554; doi:10.1007/s11325-022-02731-x)
Supplement: Supplementary file 1 — Supplementary file1 (DOCX 861 KB) [file 11325_2022_2731_MOESM1_ESM.docx]

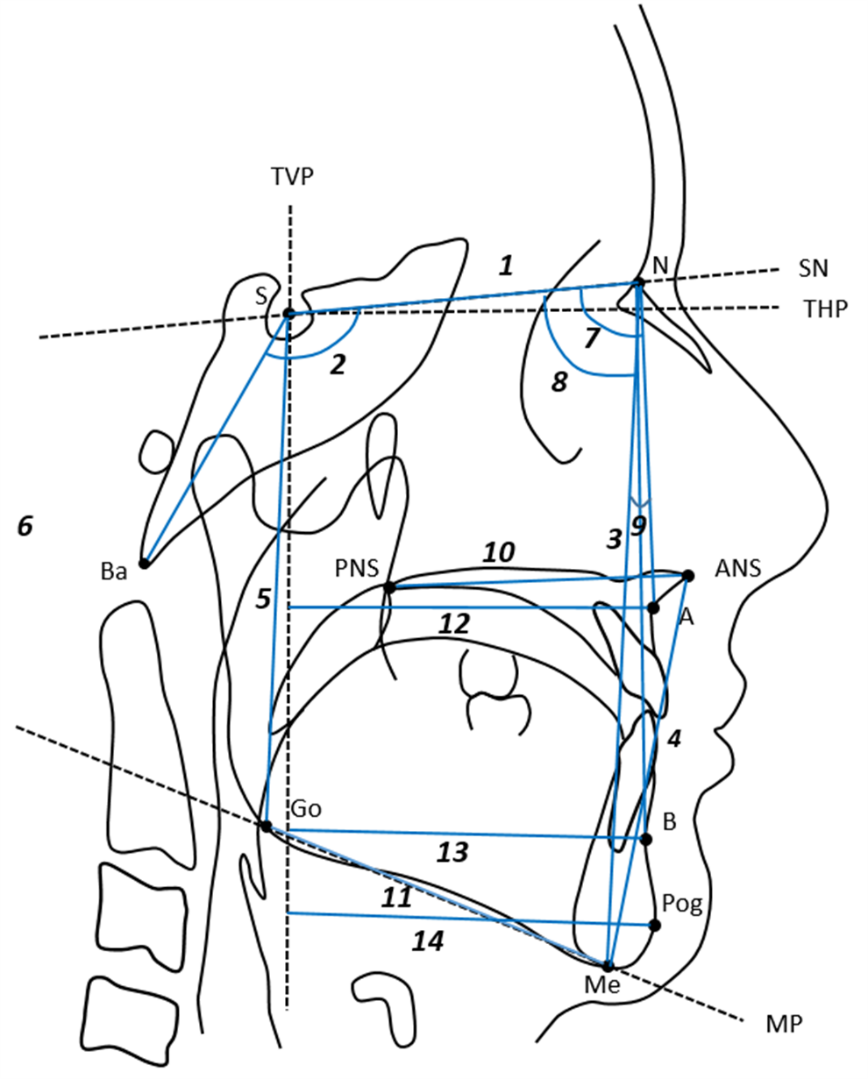


**e-Fig. 1 - Landmarks, reference lines, and the corresponding hard tissue variables used in the study.** Landmarks: A, A-point (subspinale); ANS, anterior nasal spine; B, B-point (supramentale); Ba, basion; Go, gonion; Me, menton; N, nasion; PNS, posterior nasal spine; Pog, pogonion; S, sella. Reference lines: MP, mandibular plane; SN, sella-nasion line; THP, true horizontal plane, plane through point S at 7° clockwise from SN plane; TVP, true vertical plane, plane through point S perpendicular to THP. Hard tissue variables: 1, S-N; 2, N-S-Ba; 3, ATFH (anterior total face height, N-Me); 4, ALFH (anterior lower face height, ANS-Me); 5, PTFH (posterior total face height, S-Go); 6, MP-SN; 7, SNA; 8, SNB; 9, ANB; 10, maxillary length (ANS-PNS); 11, mandibular corpus length (Go-Me); 12, A-TVP; 13, B-TVP; 14, Pog-TVP


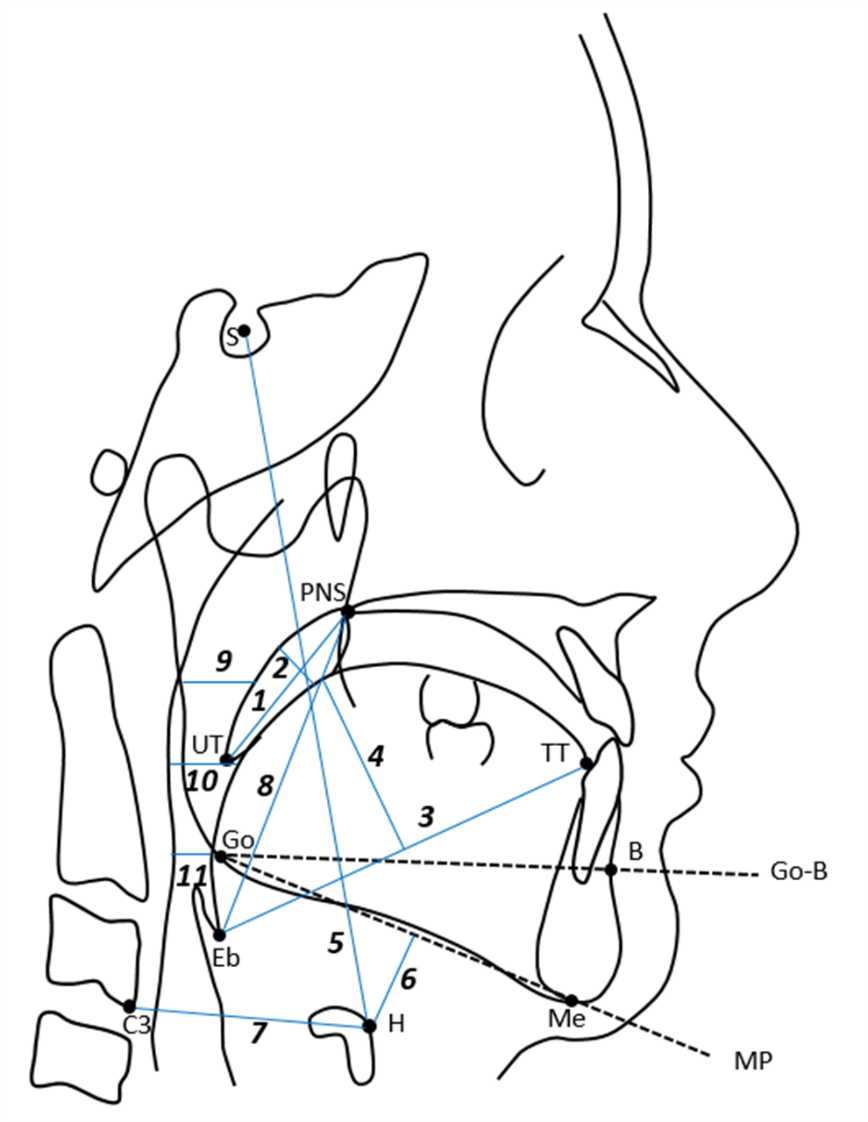


**e-Fig. 2 - Landmarks, reference lines, and the corresponding soft tissue variables used in the study.** Landmarks: B, B-point (supramentale); C3, the most anterior-inferior point of the third cervical vertebra; Eb, epiglottis base; Go, gonion; H, hyoid point; Me, menton; UT, uvula tip; PNS, posterior nasal spine; TT, tongue tip. Reference lines: Go-B, plane between Go and B; MP, mandibular plane. Soft tissue variables: 1, SPL (soft palate length); 2, SPT (soft palate thickness); 3, TGL (tongue length); 4, TGH (tongue height); 5, H-S; 6, H-MP; 7, H-C3; 8, UAL (upper airway length); 9, SPAS (superior posterior airway space); 10, MAS (middle airway space); 11, IAS (inferior airway space)

**e-Table 1. Preoperative and postoperative polysomnographic values and upper airway measurements in responders and non-responders**

| **Variables** | **Responder (n=67)** | **Non-responder (n=33)** | ***P*** |
| --- | --- | --- | --- |
| **Polysomnographic variables** | | | |
| Preop AHI (events/h) | 51.8 (37.1-68.6) | 51.6 (35.2 – 69.3) | 0.474 |
| Postop AHI (events/h) | 8.3 (4.5-13.0) | 33.0 (23.0 -42.9) | <0.001* |
| Preop ODI 3% (events/h) | 48.7 (35.3-68.9) | 57.0 (29.5-66.0) | 0.866 |
| Postop ODI 3% (events/h) | 11.2 (9.2-20.7) | 33.6 (25.8-50.3) | <0.001* |
| Preop LSAT (%) | 79 (71.0-84.0) | 80 (76.0-85.0) | 0.236 |
| Postop LSAT (%) | 87.5 (82.0-89.3) | 85.0 (82.0-87.0) | 0.019* |
| **Upper airway measurements** | | | |
| Preop UAL (mm) | 76.8 ± 6.4 | 78.8 ± 7.7 | 0.249 |
| Postop UAL (mm) | 75.1 ± 7.5 | 77.5 ± 9.5 | 0.189 |
| Preop SPAS (mm) | 7.3 (5.5-9.2) | 8.8 (7.6-11.0) | 0.002* |
| Postop SPAS (mm) | 12.5 (10.4-15.3) | 14.0 (11.7-16.1) | 0.143 |
| Preop MAS (mm) | 10.0 (7.9-12.0) | 10.8 (8.0-13.5) | 0.172 |
| Postop MAS (mm) | 14.9 (12.8-18.4) | 17.4 (12.8-20.0) | 0.202 |
| Preop IAS (mm) | 8.4 (6.7-11.5) | 8.9 (6.5-11.3) | 0.625 |
| Postop IAS (mm) | 13.6 (10.9-16.0) | 15.3 (11.5-17.5) | 0.266 |

Data presented as mean ± standard deviation or median (Q1-Q3)

*Statistically significant difference responders versus non-responders (*P* value < 0.05)

AHI, apnea hypopnea index; IAS, inferior airway space; LSAT, lowest oxygen saturation; MAS, middle airway space; n, number of patients; ODI 3%, 3% oxygen desaturation index; Postop, postoperative; Preop, preoperative; SPAS, superior posterior airway space; UAL, upper airway length.

**e-Table 2. Univariate analysis of patient-related, polysomnographic, cephalometric, and surgical variables for predicting surgical response to maxillomandibular advancement**

| **Variable** | **Coefficient B** | **SE** | **OR (95%CI)** | ***P*** |
| --- | --- | --- | --- | --- |
| **Patient-related variables** | | | | |
| Age (years) | -0.041 | 0.023 | 0.959 (0.917-1.003) | 0.070 |
| Gender |  |  |  |  |
| Female | Ref. |  |  |  |
| Male | -0.299 | 0.575 | 0.742 (0.240-2.291) | 0.604 |
| BMI (kg/m^2^) | -0.005 | 0.051 | 0.996 (0.901-1.100) | 0.930 |
| ASA-score |  |  |  |  |
| I | Ref. |  |  |  |
| II | -0.294 | 0.554 | 0.745 (0.251-2.209) | 0.596 |
| III | -0.754 | 0.648 | 0.471 (0.132-1.676) | 0.245 |
| Hypertension |  |  |  |  |
| Absence | Ref. |  |  |  |
| Presence | -1.307 | 0.447 | 0.271 (0.113-0.650) | 0.003 |
| CVD |  |  |  |  |
| Absence | Ref. |  |  |  |
| Presence | -1.465 | 0.454 | 0.231 (0.095-0.563) | 0.001 |
| DM |  |  |  |  |
| Absence | Ref. |  |  |  |
| Presence | 0.118 | 0.643 | 1.125 (0.319-3.963) | 0.855 |
| COPD |  |  |  |  |
| Absence | Ref. |  |  |  |
| Presence | -0.319 | 0.939 | 0.727 (0.115-4.574) | 0.734 |
| Previous upper airway surgery |  |  |  |  |
| Absence | Ref. |  |  |  |
| Presence | 0.038 | 0.435 | 1.038 (0.443-2.434) | 0.931 |
| Lost teeth |  |  |  |  |
| 0-4 lost teeth | Ref. |  |  |  |
| 5-8 lost teeth | -0.555 | 0.655 | 0.574 (0.159-2.074) | 0.397 |
| 9-31 lost teeth | -0.852 | 0.692 | 0.427 (0.110-1.657) | 0.219 |
| 32 lost teeth | -1.034 | 0.780 | 0.356 (0.077, 1.640) | 0.185 |
| **Polysomnographic variables** | | | | |
| AHI (events/h) | 0.007 | 0.010 | 1.007 (0.987-1.028) | 0.470 |
| CAI (events/h) | -0.191 | 0.080 | 0.826 (0.707-0.966) | 0.017 |
| MAI (events/h) | -0.013 | 0.016 | 0.987 (0.957-1.018) | 0.408 |
| Positional/non-positional OSA |  |  |  |  |
| Non-positional OSA | Ref. |  |  |  |
| Positional OSA | 0.216 | 0.476 | 1.241 (0.489-3.154) | 0.650 |
| ODI 3% (events/h) | 0.002 | 0.011 | 1.002 (0.980-1.025) | 0.864 |
| LSAT (%) | -0.033 | 0.026 | 0.967 (0.919-1.018) | 0.967 |
| **Cephalometric variables** | | | | |
| *Cranial base* |  |  |  |  |
| S-N (mm) | -0.049 | 0.059 | 0.952 (0.848-1.069) | 0.404 |
| N-S-Ba (degree) | 0.002 | 0.005 | 1.002 (0.993-1.012) | 0.627 |
| **Variable** | **Coefficient B** | **SE** | **OR (95%CI)** | ***P*** |
| *Face height* |  |  |  |  |
| ATFH (mm) | -0.035 | 0.027 | 0.966 (0.917-1.018) | 0.197 |
| ALFH (mm) | -0.038 | 0.030 | 0.963 (0.907-1.022) | 0.212 |
| PTFH (mm) | -0.036 | 0.030 | 0.964 (0.910-1.022) | 0.221 |
| MP-SN (degree) | 0.001 | 0.025 | 1.001 (0.954-1.050) | 0.979 |
| *Maxilla and mandible* |  |  |  |  |
| SNA (degree) | 0.018 | 0.060 | 1.019 (0.906-1.145) | 0.757 |
| SNB (degree) | -0.076 | 0.053 | 0.927 (0.836-1.028) | 0.150 |
| ANB (degree) | 0.144 | 0.074 | 1.155 (1.000-1.334) | 0.051 |
| ANS-PNS (mm) | 0.020 | 0.060 | 1.020 (0.907-1.147) | 0.742 |
| Go-Me (mm) | -0.044 | 0.036 | 0.957 (0.892-1.027) | 0.225 |
| *Soft palate* |  |  |  |  |
| SPL (mm) | -0.020 | 0.033 | 0.981 (0.919-1.047) | 0.557 |
| SPT (mm) | -0.086 | 0.086 | 0.918 (0.775-1.086) | 0.318 |
| *Tongue* |  |  |  |  |
| TGL (mm) | -0.029 | 0.033 | 0.971 (0.911-1.035) | 0.371 |
| TGH (mm) | 0.076 | 0.054 | 1.079 (0.971-1.199) | 0.158 |
| *Pharyngeal dimensions and hyoid bone position* |  |  |  |  |
| UAL (mm) | -0.038 | 0.033 | 0.963 (0.903-1.027) | 0.248 |
| SPAS (mm) | -0.242 | 0.083 | 0.785 (0.666-0.924) | 0.004 |
| MAS (mm) | -0.105 | 0.077 | 0.900 (0.773-1.047) | 0.173 |
| IAS (mm) | -0.036 | 0.072 | 0.965 (0.837-1.112) | 0.621 |
| H-S (mm) | -0.031 | 0.024 | 0.969 (0.924-1.016) | 0.196 |
| MP-H (mm) | -0.016 | 0.040 | 0.984 (0.910-1.063) | 0.678 |
| H-C3 (mm) | -0.066 | 0.042 | 0.936 (0.863-1.015) | 0.110 |
| **Surgical variables** | | | | |
| Advancement degree of A-point (mm) | -0.068 | 0.097 | 0.934 (0.772-1.130) | 0.481 |
| Advancement degree of B-point (mm) | 0.024 | 0.057 | 1.024 (0.916-1.146) | 0.675 |
| Advancement degree of Pog (mm) | -0.005 | 0.046 | 0.995 (0.910-1.088) | 0.908 |
| Counterclockwise rotation (n, %) |  |  |  |  |
| Absence | Ref. |  |  |  |
| Presence | -0.311 | 0.476 | 0.733 (0.288-1.862) | 0.513 |

A, A-point; AHI, apnea hypopnea index; ALFH, anterior lower face height; ANS, anterior nasal spine; ASA, American Society of Anesthesiology; ATFH, anterior total face height; B, B-point; Ba, basion; BMI, body mass index; C3, the most anterior-inferior point of the third cervical vertebra; CAI, central apnea index; CI, confidence interval; COPD, chronic obstructive pulmonary disease; CVD, cardiovascular disease; DM, diabetes mellitus; Go, gonion; H, hyoid bone; IAS, inferior airway space; LSAT, lowest oxygen saturation; MAI, mixed apnea index; MAS, middle airway space; Me, menton; mm, millimeter; MP, mandibular plane; N, nasion; ODI 3%, 3% oxygen desaturation index; OR, odds ratio; OSA, obstructive sleep apnea; PNS, posterior nasal spine; PTFH, posterior total face height; Ref., reference category; S, sella; SE, standard error; SPAS, superior posterior airway space; SPL, soft palate length; SPT, soft palate thickness; TGL, tongue length; TGH, tongue height; UAL, upper airway length.
